# ORAL PLENARY SESSION 1

**DOI:** 10.1002/pmf2.12001

**Published:** 2025-01-05

Abstracts 1 – 8

THURSDAY

January 30

8 AM – 10 AM

Aurora Ballroom

MODERATORS

Cynthia Gyamfi‐Bannerman, MD, MS

Anthony C. Sciscione, DO

